# Assessment of the therapeutic efficacy of *Holothuria polii* extract during the muscular phase of *Trichinella spiralis* infection in albino mice

**DOI:** 10.1038/s41598-026-60075-y

**Published:** 2026-07-05

**Authors:** Salwa A. El-Saidy, Asmaa Sabry, Gihan M. El-Khodary, Amal A. A. Hassan, Dina I. Elgendy, Yasmeen M. Gawaan

**Affiliations:** 1 Zoology Department, Faculty of Science, Damanhour University, Damanhour, 22511 Egypt; 2 Medical Parasitology Department, Faculty of Medicine, Tanta University, Tanta, Egypt

**Keywords:** Marine invertebrates, Mice, Oxidative stress, Antioxidants, Histology, Immunohistochemistry, Biotechnology, Diseases, Drug discovery, Microbiology, Zoology

## Abstract

Marine invertebrates are an important source of bioactive natural products with potential therapeutic applications, owing to their chemically diverse defense mechanisms. Trichinellosis is a widespread food-borne zoonosis caused by *Trichinella spiralis (T. spiralis)*. Albendazole (ABZ) is the main treatment, but its efficacy against encysted and newborn larvae is limited. This limitation, together with emerging resistance, has prompted the search for safer alternative antiparasitic agents, particularly natural compounds. *Holothuria polii* (*H. polii*) is a bioactive-rich sea cucumber with promising therapeutic applications. The current study was designed to assess the anti-*T. spiralis* effectiveness of *H. polii* extract (HPE), for the first time, against the muscular phase of experimental trichinellosis, either alone or in combination with ABZ, and to explore its prospective application as an adjunct to ABZ therapy to enhance treatment outcomes and tissue recovery. To fulfill this aim, mice were distributed at random into five experimental groups, as follows: a control group and four *T. spiralis*-infected groups: untreated, ABZ-treated, HPE-treated, and combined therapy-treated. The treatment with HPE, alone or combined with ABZ, reduced larval burden and improved host muscular tissue health by reducing oxidative stress, enhancing antioxidant defenses, and restoring serum liver and muscular enzyme activities to their normal values. Histopathological examination of the treated groups revealed preservation of the skeletal and diaphragmatic muscular architecture, reduced inflammatory cell infiltration, and degeneration of larval capsules, particularly with combined therapy. These findings were accompanied by downregulation of cyclooxygenase-2 (COX-2) and vascular endothelial growth factor (VEGF) immune expressions in both skeletal and diaphragmatic muscles compared with the untreated and ABZ-treated groups, indicating reduced inflammation and angiogenesis. The present study concluded that HPE exhibits diverse and efficacious activity against trichinellosis and may be regarded as a promising adjuvant treatment alongside ABZ for *T. spiralis* infection. However, further studies are required to validate its potential efficacy in clinical applications.

**Supplementary Information:**

The online version contains supplementary material available at 10.1038/s41598-026-60075-y.

## Introduction

Marine invertebrates are an important source of bioactive natural products with biomedical potential^1^. Among them, sea cucumbers have gained attention for their broad-spectrum bioactivities, particularly antiparasitic properties^2^. These activities are mainly attributed to saponins, the principal secondary metabolites in sea cucumber tissues, which contribute to their therapeutic potential against parasitic diseases through their membranolytic properties^3,4^.

Trichinellosis is a globally distributed foodborne zoonotic disease caused by *Trichinella spiralis (T. spiralis)*, transmitted to humans mainly through feeding on raw or undercooked pork containing encysted larvae^5^. After ingestion, larvae metamorphose into adults in the small intestine and subsequently release newborn larvae that migrate to striated muscles, initiating the muscular phase of infection, characterized by myalgia, periorbital edema, inflammation, and muscle damage^6,7^. Larval migration and excretory-secretory products induce inflammatory and allergic reactions, accompanied by lymphocyte and eosinophil infiltration and increased leakage of muscular enzymes into the circulation^8^. In the striated muscular tissues, larvae transform infected myocytes into nurse cells that support long-term parasite survival through encapsulation, angiogenesis, and immune evasion^9^. Encystment occurs preferentially in highly active muscles, where larvae become infective and may persist for years^10^. Hypoxia-induced vascular endothelial growth factor (VEGF) expression promotes angiogenesis around nurse cells, ensuring nutrient supply for larval maintenance^11,12^. Moreover, excessive production of reactive oxygen species (ROS) during infection contributes to lipid peroxidation and muscle injury^13,14^. Histopathological alterations include myofiber degeneration, inflammatory infiltration, edema, and collagen capsule formation around nurse cell–larva complexes, which may progress to fibrosis and calcification during chronic infection^15,16^.

Albendazole (ABZ) is a broad-spectrum benzimidazole anthelmintic drug widely used to treat *T. spiralis* infections because of its proven efficacy, oral administration, low cost, and favorable therapeutic index^6,17^. It is particularly effective during the early stage of infection, reducing adult worm burden and intestinal larval production^17,18^. Its antiparasitic activity is primarily attributed to impeding microtubule polymerization through selective binding to parasite β-tubulin and impairment of glucose uptake, leading to parasite starvation^19^. However, its efficacy against encysted and newborn larvae is limited^18^. Furthermore, ABZ suffers from poor solubility, low bioavailability, reduced intestinal absorption, and the emergence of drug resistance^20,21^. Its use may also be associated with gastrointestinal, hepatic, hematological, and neurological adverse effects at high doses^22^. Therefore, its use during pregnancy is generally avoided, particularly in the first trimester, due to potential teratogenic and fetotoxic risks. In addition, its use in young children, especially those under two years of age, is restricted because of the risk of adverse effects^23^. These limitations have driven interest in safer and more effective natural alternatives, particularly from marine invertebrates that have shown promising antiparasitic activities^2^.

*Holothuria polii(*H. polii)^24^ is a dominant holothurian species along the Egyptian Mediterranean coast, characterized by a dark-brown, cylindrical body with a thick integument containing calcareous ossicles^25,26^. Its biological activities are mainly attributed to bioactive metabolites present in its crude extract^26,27^. Previous studies have reported antiparasitic, antimicrobial, antitumor, and anti-inflammatory properties of *H. polii* extract (HPE), largely due to triterpene glycoside saponins such as echinosides A and B^28–31^. The antitumor activity of its aqueous fraction has been linked to inhibition of cell proliferation, suppression of epithelial–mesenchymal transition markers, and modulation of inflammatory mediators, resulting in reduced tumor progression and metastasis^31^. In addition, ethanolic HPE demonstrated significant activity against adult worms of *T. spiralis*^26^ and *Schistosoma mansoni*^32^. Accordingly, the current study was designed to evaluate the therapeutic effectiveness of HPE against the muscular phase of experimental trichinellosis in male Swiss albino mice and to assess its potential as an adjunctive therapy to ABZ. To the best of our knowledge, this is the first study to evaluate HPE in this context, aiming to enhance treatment outcomes and tissue recovery.

## Materials and methods

### Sampling and identification of ***H. polii***

**Fig. 1:**
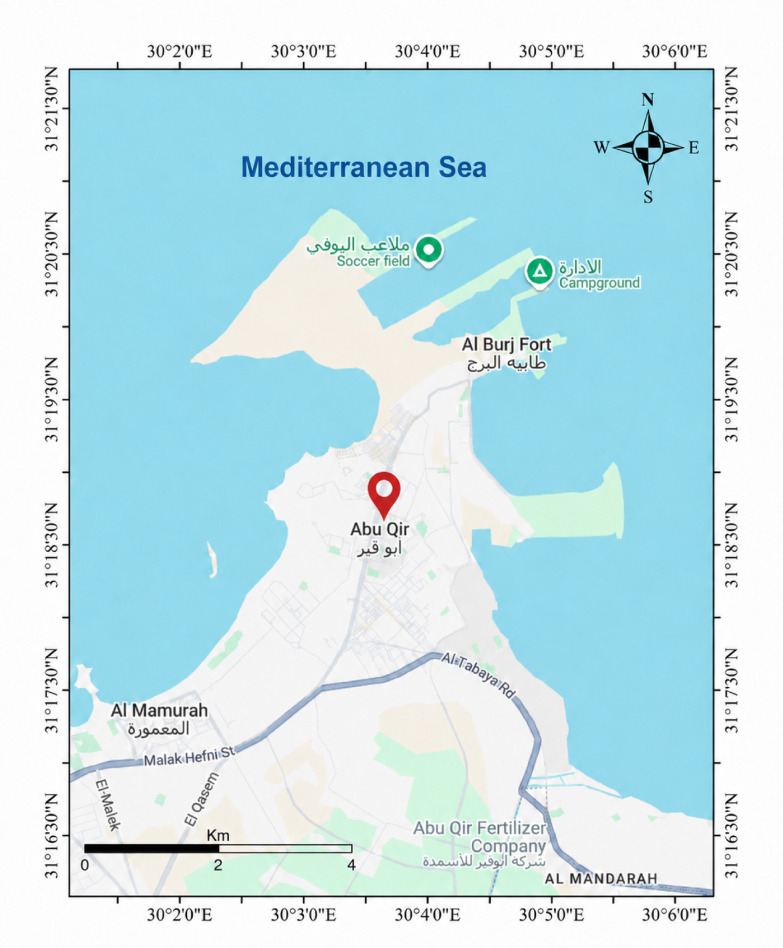
A map of the *H. polii* sampling site, Abu Qir (red dot), on the Egyptian Mediterranean Sea coast, Alexandria. The map was created using ArcGIS Desktop version 10.8 (Esri, Redlands, CA, USA; https://desktop.arcgis.com/).

*H. polii* specimens were collected from Abu Qir, Alexandria, Egypt (31°18′45.96″N, 30°03′38.16″E, Fig. 1), following local regulations. Species identification was based on morphological traits and ossicle shape, as previously described^25,26^. In the laboratory, samples were washed and eviscerated, and the body wall was chopped and stored at − 20 °C until extraction.

### Preparation of ***H. polii*** ethanolic extract

The ethanolic HPE was prepared from the body wall following Mona et al.^32^. The tegument homogenate was prepared using 95% ethanol at a 1:4 weight-to-volume ratio and incubated in the dark at ambient temperature for three days with gentle agitation. The homogenate was then filtered, concentrated under reduced pressure, and lyophilized to yield 0.6% of the initial weight. Finally, the dried extract was preserved at − 80 °C until use.

### Preparation of the inoculum of ***T. spiralis*** larvae

The isolate of *T. spiralis* (ISS6158) was initially recovered from infected pork meat at the Cairo abattoir and preserved in the laboratory *via* serial passages in mice. Five weeks post-infection, the mouse tissues were digested with artificial gastric juice (1% pepsin and 1% concentrated hydrochloric acid in 200 ml of warm distilled water) at 37 °C for 2 h with continuous stirring and then filtered. Larvae were recovered by sedimentation, extensively rinsed with phosphate-buffered saline (PBS), and finally enumerated microscopically using a McMaster counting chamber^33,34^.

### In vivo experimental design and dosage regimen

Forty-five male Swiss albino mice, 6–8 weeks old and 25–30 g in weight, were acquired from the Theodore Bilharz Research Institute, Giza, Egypt. Mice were maintained in polypropylene cages in the Zoology Department’s animal house, Faculty of Science, Damanhour University, under standard laboratory conditions (five mice per cage, 25 °C, and a 12 h light/dark cycle) with adequate ventilation and unrestricted access to standard food and potable water throughout the experimental period. Mice were acclimatized for seven days before the initiation of the experiment. Mice were randomly assigned to five experimental groups as detailed below:

*Control group (GI).* Five mice were neither infected nor treated.

*Infected and untreated group (GII).* Ten mice were infected orally with 200 *T. spiralis* larvae in 0.25 ml of distilled water/mouse, based on El-Hamed et al.’s study^35^, and administered no treatment.

*Infected and ABZ-treated group (GIII).* Ten *T. spiralis*-infected mice were administered an oral dose of 50 mg ABZ/kg body weight (b.w.)/day, following Salama et al.^7^. ABZ [Alzental^®^ suspension with a concentration of 20 mg/ml; Egyptian International Pharmaceutical Industries Co. (EIPICO)] was diluted with sterile distilled water to obtain the required dose.

*Infected and HPE-treated group (GIV).* Ten *T. spiralis*-infected mice were administered an oral dose of 5.4 mg HPE/kg b.w./day, a dosage selected based on the anti-schistosomal study by Mona et al.^32^.

*Infected and combined therapy-treated group (GV).* Ten *T. spiralis*-infected mice were orally administered both ABZ and HPE at the identical dosages as the preceding groups.

Treatment was administered on the 31^st^ day post-infection (dpi) and continued for 14 days^36^. The animals were monitored daily throughout the experimental period for clinical signs, including changes in activity, behavior, fur condition, and posture, as well as mortality. Following overnight fasting, mice were deeply anesthetized intraperitoneally with sodium pentobarbital (40 mg/kg b.w.), euthanized by cervical dislocation, and dissected to assess the treatment efficacy against *T. spiralis* muscle larvae.

### Ethics approval

All procedures were conducted in accordance with the ARRIVE guidelines and institutional regulations for the care and use of laboratory animals, which were approved by the Ethics Committee of the Faculty of Science at Damanhour University in Egypt (Ethical Approval Code: DMU-SCI-CSRE-260302).

### Evaluation of the treatment’s efficiency during the muscular phase

#### Isolation and counting of total**T. spiralis** encysted larvae

The muscle larval count of infected mice was determined in the same way as that described in the muscle larval preparation method for infection^34^.

### Biochemical analyses

#### Measurement of lipid peroxidation and antioxidant markers

A skeletal muscular tissue sample was excised, washed with ice-cold saline, finely chopped, and weighed. It was then homogenized in 0.1 M Tris-EDTA buffer (pH 7.4; 1:10 *w/v*). The homogenate was centrifuged at 4000 rpm for 15 min at 4 °C, and the supernatant was preserved at − 80 °C for further analyses. The lipid peroxidation end product and antioxidant biomarkers were measured using Biodiagnostic Company kits: malondialdehyde (MDA; catalog number MD 25 29) and antioxidant biomarkers [reduced glutathione (GSH; catalog number GR 25 11) & catalase (CAT; catalog number CA 25 17)]. MDA level was assessed by the thiobarbituric acid colorimetric assay^37^ (nmol/g tissue); GSH level was determined using the 5,5′-dithiobis(2-nitrobenzoic acid) colorimetric methodology of Beutler et al.^38^ (mg/g tissue); and CAT activity was measured according to Aebi’s method^39^ based on residual hydrogen peroxide (H₂O₂) detection (U/g tissue).

#### Determination of enzyme activities in blood serum

Blood samples were obtained *via* heart puncture into sterile tubes, allowed to clot at ambient temperature for 30 min, and centrifuged at 3000 rpm for 10 min. The obtained sera were retained at − 20 °C until biochemical analyses. Serum activities of alanine aminotransferase (ALT; catalog number 265 001) and aspartate aminotransferase (AST; catalog number 291 000) were determined using Spectrum Diagnostics Company kits (Cairo, Egypt) according to the kinetic method of Young^40^. Alkaline phosphatase (ALP; catalog number AP 10 20) was determined using a Biodiagnostic Company kit (Giza, Egypt) following Belfield and Goldberg^41^, while the activities of lactate dehydrogenase (LDH; catalog number 13201) and creatine kinase (CK; catalog number 11302) were assayed using Vitro Scient Company kits (Industrial area, Basatin Al Ismailia, Egypt) according to the methods of Moss et al.^42^ and Young^40^, respectively. All enzyme activities were expressed as U/L.

### Histological study

Samples of thigh and diaphragm muscles (1–2 cm) were preserved in 10% neutral buffered formalin overnight, then dehydrated in graded ethanol, cleared with xylene, and embedded in molten paraffin wax at 58 °C. Serial 4 μm sections were prepared, mounted, deparaffinized, and eventually stained with hematoxylin and eosin (H&E). Slides were examined using a light microscope (Olympus CX31, Tokyo, Japan) and then photographed to evaluate histological changes^43^.

### Immunohistochemical study

Cyclooxygenase-2 (COX-2) and VEGF expressions in thigh and diaphragm muscle tissues were assessed using the methodology of Hsu et al.^44^. Paraffin sections were deparaffinized, rehydrated, and subjected to antigen retrieval in citrate buffer. Endogenous peroxidase activity was blocked with 3% H₂O₂ for 5 min. Sections were treated overnight at 37 °C with mouse anti-COX-2 monoclonal antibody (1:50; Santa Cruz Biotechnology, Inc., USA; catalog number sc-19999) and/or rabbit anti-VEGF polyclonal antibody (1:50; Boster Biological Technology, Valley Ave, Pleasanton, CA; catalog number PA1080). After PBS washing, sections were treated with a biotinylated goat anti-mouse IgG antibody for 30 min, followed by incubation with streptavidin-conjugated peroxidase for 30 min at 37 °C. Immunoreactivity was visualized using a 3,3-diaminobenzidine tetrahydrochloride solution for 5 min and counterstained with Mayer’s hematoxylin; positively expressed cells showed brown cytoplasmic staining. Expression levels were quantified in random non-overlapping fields using ImageJ Version 1.47 software (RRID: SCR_003070)^45^.

### Data analysis

For each quantitative analysis, data are presented as the mean ± standard deviation (SD) of three independent biological replicates per group. Statistical analysis was conducted using one-way analysis of variance (ANOVA) followed by Tukey’s Honest Significant Difference (Tukey’s HSD) test for multiple comparisons using the statistical package for the social sciences (IBM-SPSS v20) software, with statistical significance established at *p* ≤ 0.05^46,47^.

## Results

### Clinical observations

During the experimental period, no overt clinical signs or mortalities were observed in mice across all groups, especially in the infected, untreated group, despite the successful establishment of the infection with 200 *T. spiralis* larvae per mouse. All the experimental animals appeared to have normal activity with normal intake of food and water.

### Parasitological findings

#### Count of **T. spiralis** larvae in the muscles of the different infected mice groups

**Fig. 2:**
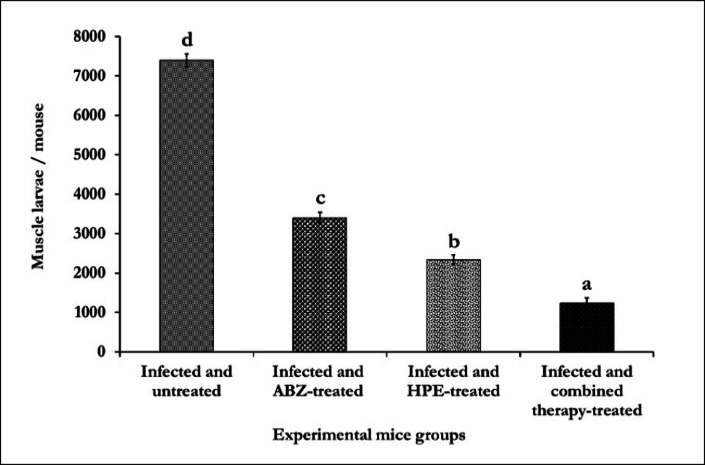
Count of *T. spiralis* larvae in the muscles of the different infected mice groups. The data are presented as the mean ± SD of three independent biological replicates per group. Different letters ^(a, b, c, and d)^ demonstrate significant differences (*p* ≤ 0.05, one-way ANOVA).

All treated groups showed highly significant reductions (*p* < 0.001) in muscle larval count relative to the infected, untreated group. The combined therapy achieved the highest reduction percentage (83.33%), followed by HPE treatment (68.47%), whereas ABZ treatment showed the lowest reduction percentage (54.04%) (Fig. 2 and Table S1).

### Biochemical findings

#### Oxidative stress and antioxidant markers in the skeletal muscular tissues of the different mice groups

**Fig. 3:**
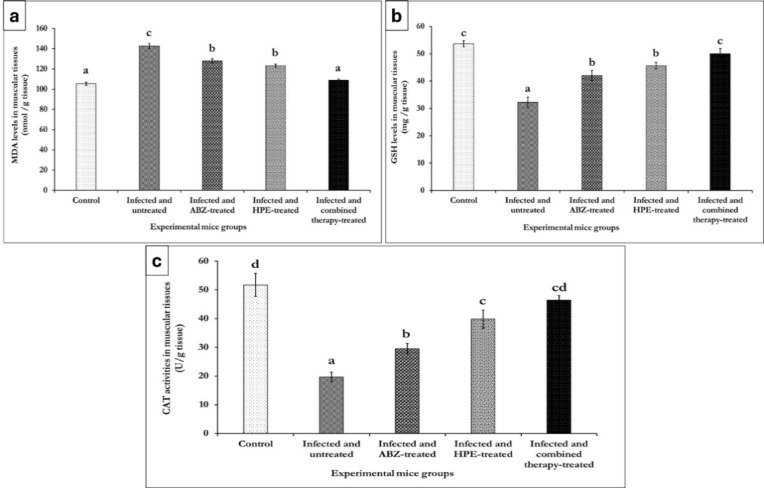
Oxidative stress and antioxidant markers in the skeletal muscular tissue of the different mice groups. **(a)** malondialdehyde (MDA), **(b)** reduced glutathione (GSH), and **(c)** catalase (CAT). The data are presented as the mean ± SD of three independent biological replicates per group. Different letters ^(a, b, c, and d)^ demonstrate significant differences (*p* ≤ 0.05, one-way ANOVA).

Oxidative stress markers were significantly altered in the infected, untreated group compared with the control group, as evidenced by a highly significant elevation (*p* < 0.001) in MDA level and highly significant decreases (*p* **˂** 0.001) in GSH level and CAT activity relative to the control group. Treatment with either ABZ or HPE significantly ameliorated these alterations (*p* < 0.01 and *p* < 0.001, respectively), although significant differences (*p* < 0.001 and *p* < 0.01, respectively) from the control group remained. In contrast, combined therapy restored these parameters to near-normal values, showing a highly significant improvement (*p* < 0.001) compared with the infected, untreated group and no significant difference (*p* > 0.05) from the control group (Fig. 3 and Table S2).

#### Enzyme activities in the blood serum of the different mice groups

**Fig. 4:**
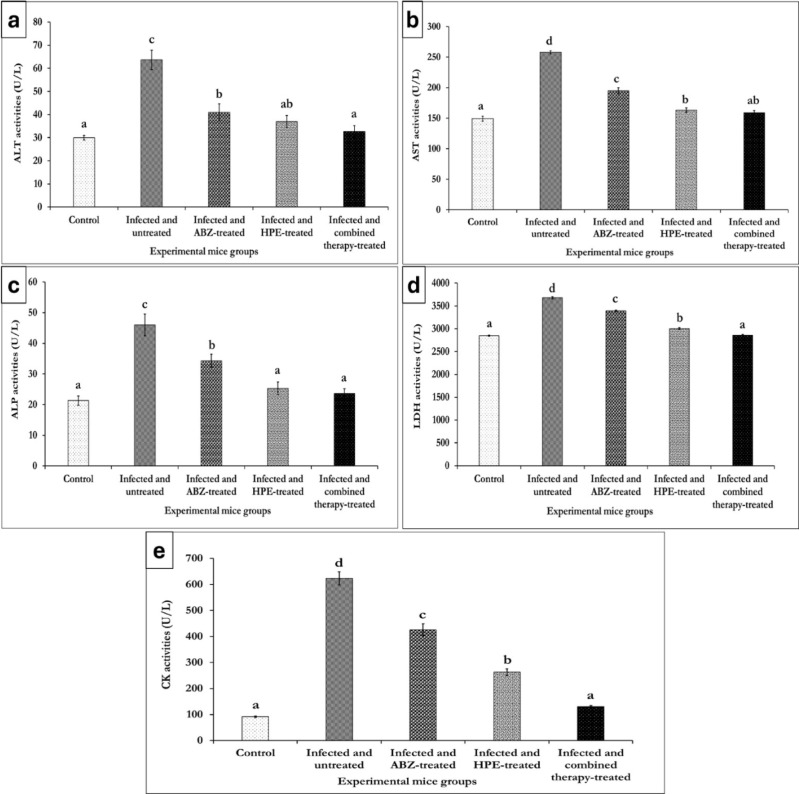
Serum enzymatic activities in the different mice groups. **(a)** alanine aminotransferase (ALT), **(b)** aspartate aminotransferase (AST), **(c)** alkaline phosphatase (ALP), **(d)** lactate dehydrogenase (LDH), and **(e)** creatine kinase (CK). The data are presented as the mean ± SD of three independent biological replicates per group. Different letters ^(a, b, c, and d)^ demonstrate significant differences (*p* ≤ 0.05, one-way ANOVA).

Serum enzyme activities were markedly altered following infection. The infected, untreated group showed highly significant elevations (*p* < 0.001) in ALT, AST, ALP, LDH, and CK activities relative to the control group. Treatment with ABZ, HPE, or their combination significantly ameliorated these alterations, resulting in highly significant reductions (*p* ≤ 0.001) in all measured enzyme activities relative to the infected, untreated group. Among the treated groups, combined therapy produced the greatest improvement with highly significant decreases (*p* **˂** 0.001) in all studied enzymes relative to the infected, untreated group, restoring their activities to levels that did not differ significantly (*p* > 0.05) from those of the control group. In contrast, ABZ- and HPE-treated groups still exhibited significant increases (*p* **˂** 0.01 and *p* **˂** 0.001) in some enzyme activities compared with the control group (Fig. 4 and Table S3).

### Histological findings

#### Skeletal muscle observations of the different mice groups

**Fig. 5:**
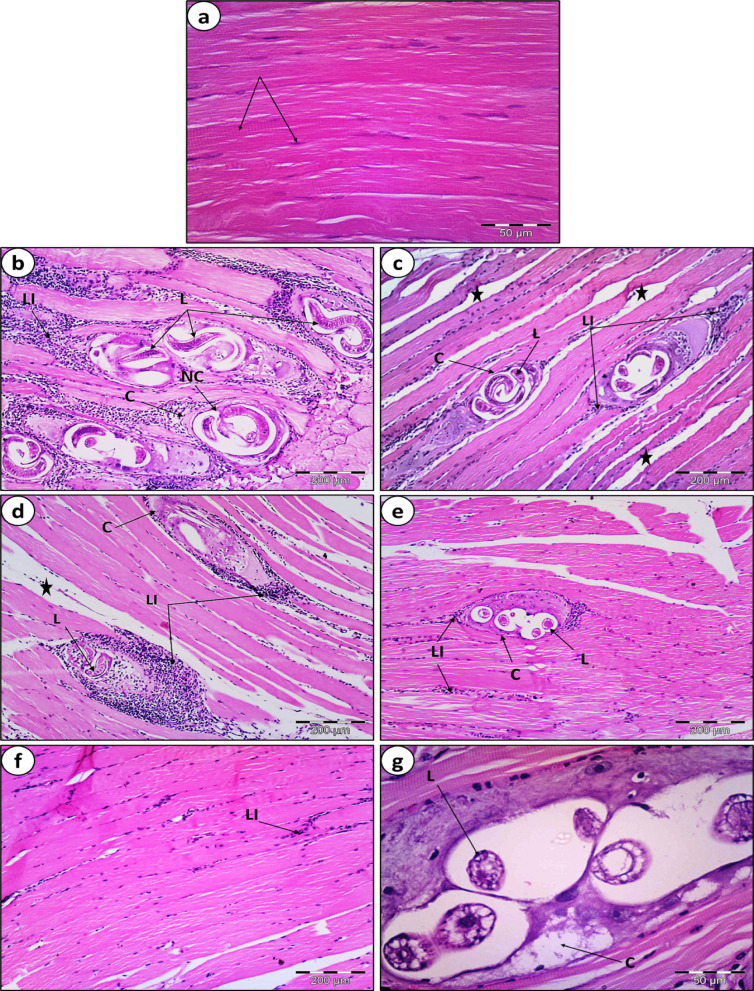
Photomicrographs of H&E-stained skeletal muscle sections of **(a)** a control mouse showing normal elongated skeletal muscle fibers (arrows) characterized by acidophilic cytoplasm, numerous peripheral basophilic nuclei, and transverse striations, **(b & c)** an infected mouse receiving no treatment, showing an alteration in architecture of the muscle fibers with a large number of encysted *T. spiralis* larvae (L), each one in a nurse cell (NC) and collagen capsule (C) deposited within the muscle bundles, severe inflammatory leucocytic infiltration (LI) mainly surrounding the encysted larvae at both poles of the capsules and between the degenerative skeletal muscle bundles, and appearance of separations between muscle bundles (stars), **(d)** an infected mouse receiving ABZ, showing fewer numbers of the encysted larvae (L) in comparison to the infected, untreated mouse with incomplete degeneration of capsules (C), marked inflammatory leucocytic infiltrations (LI) surrounding them, and appearance of separation between muscle bundles (star), **(e)** an infected mouse receiving HPE, showing a mild inflammatory leucocytic infiltrations (LI) around the capsule (C) containing *T. spiralis* larva (L) and between the skeletal muscle bundles in addition to an improvement in muscle fiber architecture, and **(f & g)** an infected mouse receiving combined therapy, showing an improvement in muscle fiber architecture with a minimal inflammatory leucocytic infiltration (LI) between muscle fibers and *T. spiralis* larva (L) inside the degenerated capsule (C).

Histological examination of the skeletal muscles revealed normal muscular architecture in the control group. In contrast, the infected, untreated group showed markedly degenerated skeletal muscle fibers with hyaline degeneration in some sections and numerous encysted *T. spiralis* larvae associated with severe inflammatory cell infiltrations around the collagen capsules and between the necrotic muscle fibers. Treatment with ABZ or HPE, alone or in combination, ameliorated these alterations to varying degrees. Treatment with ABZ reduced the parasite burden; however, a number of larval capsules and inflammatory changes persisted. HPE treatment improved muscle architecture and reduced inflammation. The combined therapy produced the most pronounced histopathological improvement, resulting in a nearly normal muscle architecture with a minimal level of inflammation between the muscle fibers, in addition to degeneration of larval capsules (Fig. 5).

#### Diaphragm muscle observations of the different mice groups

**Fig. 6:**
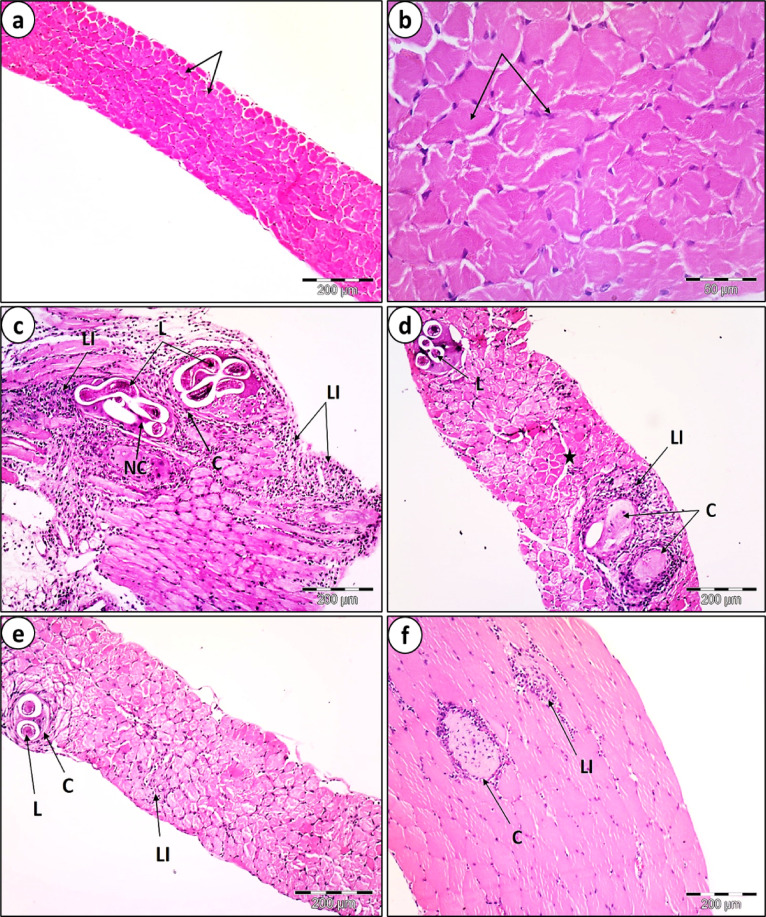
Photomicrographs of H&E-stained diaphragm muscle sections of **(a & b)** a control mouse showing normally organized diaphragm muscle fibers (arrows), distinguished by a polyhedral shape, oval peripheral nuclei, and acidophilic cytoplasm, **(c)** an infected mouse receiving no treatment, showing alterations in the diaphragm muscle fiber architecture with massive inflammatory leucocytic infiltrations (LI) mainly surrounding the encysted larvae (L), each of them appears within a nurse cell (NC) and collagen capsule (C) and is deposited in between disorganized muscle fibers, **(d)** an infected mouse receiving ABZ, showing an encysted larva (L), degenerated capsules (C) invaded and surrounded by a moderate inflammatory leucocytic infiltration (LI), and appearance of separation between muscle fibers (star), **(e)** an infected mouse receiving HPE, showing an improvement of diaphragm muscle fiber architecture with a mild inflammatory leucocytic infiltration (LI) between muscle fibers and presence of thin collagen capsule (C) around the larva (L), and **(f)** an infected mouse receiving combined therapy, showing an improvement in diaphragm muscle fiber architecture and appearance of degenerated capsule (C), surrounded by a mild inflammatory leucocytic infiltration (LI) between muscle fibers.

Histological examination of the diaphragm sections revealed normal architecture in the control group. The infected, untreated group demonstrated marked structural alterations, including inflammatory cell infiltrations and encysted larvae between disorganized muscle fibers. Treatment with ABZ or HPE resulted in partial improvement of the histological architecture, with reduced inflammatory infiltrations and occurrence of degenerative changes in the larvae and capsules. The most pronounced improvement was observed in the combination therapy group, which showed near-normal diaphragm muscle architecture and minimal inflammatory infiltrations (Fig. 6).

### Immunohistochemical findings

#### Cyclooxygenase-2 immune expressions in the skeletal and diaphragm muscle sections

**Fig. 7:**
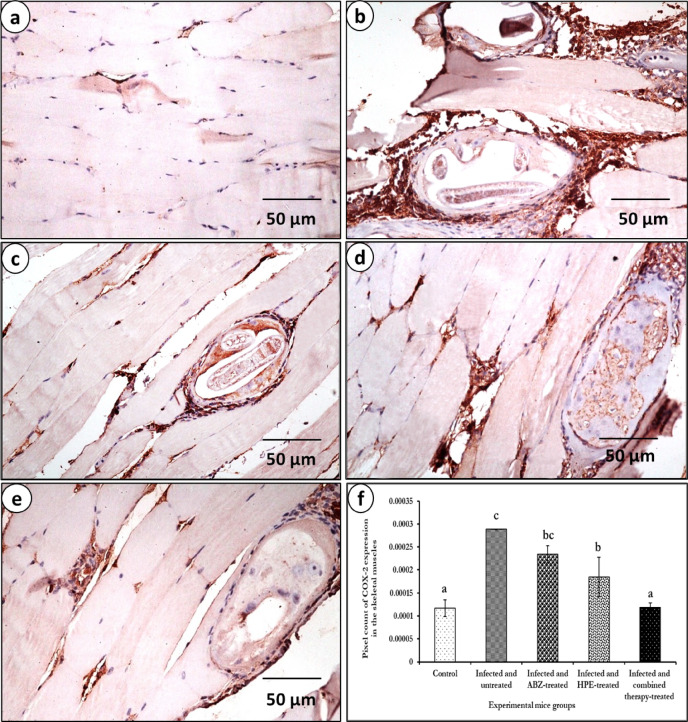
Photomicrographs of cyclooxygenase-2 (COX-2) cytoplasmic expression levels appeared as a brown color in the skeletal muscle sections of mice: **(a)** a control section demonstrating a weak positive COX-2 expression, **(b)** an infected, untreated section demonstrating a strong positive COX-2 expression primarily in the cytoplasm of the inflammatory cells between skeletal muscle fibers and around nurse cells, **(c)** an infected and ABZ-treated section demonstrating a moderate positive expression of COX-2, **(d)** an infected and HPE-treated section demonstrating a moderate positive expression of COX-2, and **(e)** an infected and combined therapy-treated section demonstrating a weak positive expression of COX-2 (COX-2 immunostain, counterstained with hematoxylin). **(f)** Statistical analysis of COX-2 cytoplasmic expression levels in the skeletal sections of the different mice experimental groups. The data are presented as the mean ± SD of three independent biological replicates per group. Different letters ^(a, b, and c)^ demonstrate significant differences (*p* ≤ 0.05, one-way ANOVA).

**Fig. 8:**
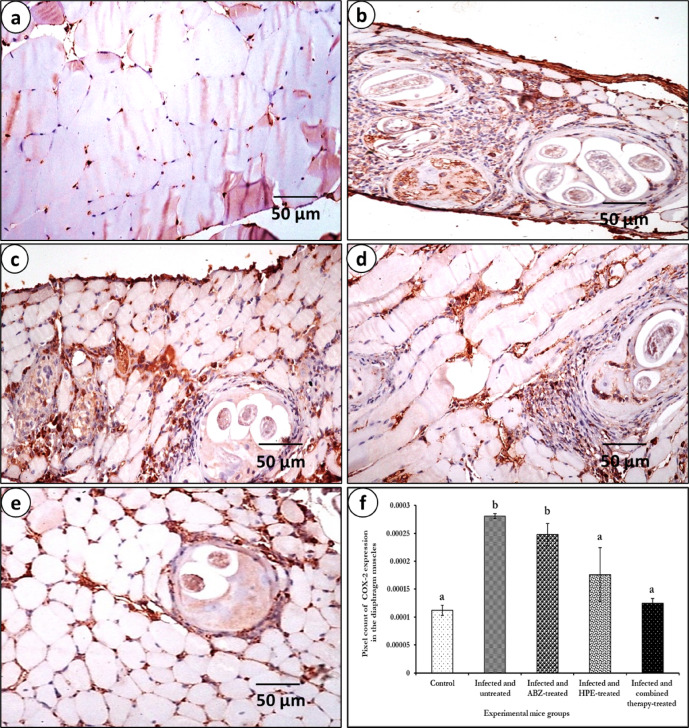
Photomicrographs of cyclooxygenase-2 (COX-2) cytoplasmic expression levels appeared as a brown color in the diaphragm muscle sections of mice: **(a)** a control section demonstrating a weak positive COX-2 expression, **(b)** an infected, untreated section demonstrating a strong positive COX-2 expression primarily in the cytoplasm of the inflammatory cells between diaphragmatic muscle fibers and around nurse cells, **(c)** an infected and ABZ-treated section demonstrating a moderate positive expression of COX-2, **(d)** an infected and HPE-treated section demonstrating a mild positive expression of COX-2, and **(e)** an infected and combined therapy-treated section demonstrating a weak positive expression of COX-2 (COX-2 immunostain, counterstained with hematoxylin). **(f)** Statistical analysis of COX-2 cytoplasmic expression levels in the diaphragm muscle sections of the different mice experimental groups. The data are presented as the mean ± SD of three independent biological replicates per group. Different letters ^(a, b, and c)^ demonstrate significant differences (*p* ≤ 0.05, one-way ANOVA).

COX-2 immunohistochemical expression was highly significantly elevated (*p* < 0.001) in both skeletal and diaphragm muscles of the infected, untreated group compared with the control group. Treatment with ABZ reduced COX-2 expression in both muscle types; however, levels remained significantly higher than those in the control group (*p* ≤ 0.001) and did not differ significantly from those in the infected, untreated group (*p* > 0.05). HPE treatment significantly decreased COX-2 expression compared with the infected, untreated group (*p* < 0.01), with expression levels approaching those of the control group, with a significant increase (*p* < 0.05) in the skeletal muscles and a non-significant increase (*p* > 0.05) in the diaphragmatic muscles. The combined therapy produced the greatest reduction in COX-2 expression in both muscle types, showing highly significant decreases (*p* < 0.001) compared with the infected, untreated group and no significant differences (*p* > 0.05) from the control group (Figs. 7 and 8 and Table S4).

#### Vascular endothelial growth factor immune expressions in the skeletal and diaphragm muscle sections

**Fig. 9:**
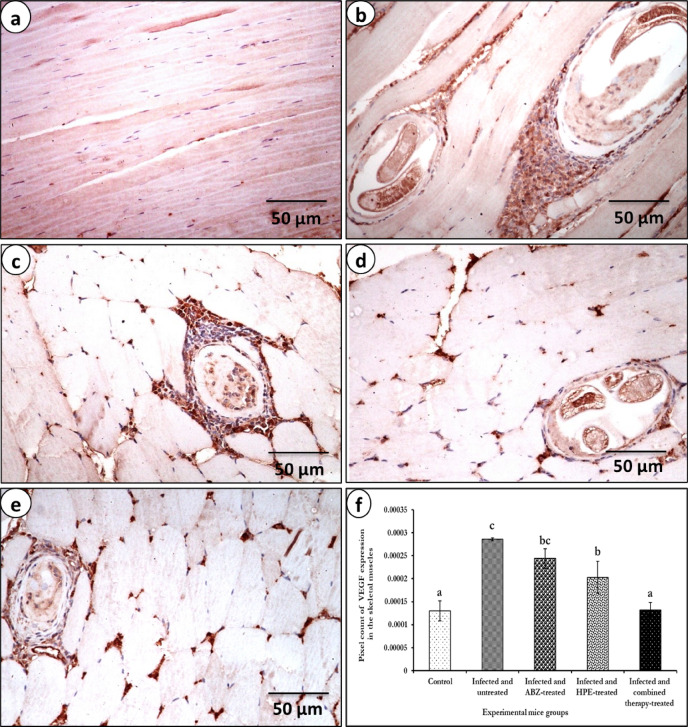
Photomicrographs of vascular endothelial growth factor (VEGF) cytoplasmic expression levels appeared as a brown color in the skeletal muscle sections of mice: **(a)** a control section demonstrating a weak positive VEGF expression, **(b)** an infected, untreated section demonstrating a strong positive VEGF expression primarily in the cytoplasm of the inflammatory cells between skeletal muscle fibers and around nurse cells, **(c)** an infected and ABZ-treated section demonstrating a moderate positive expression of VEGF, **(d)** an infected and HPE-treated section demonstrating a moderate positive expression of VEGF, and **(e)** an infected and combined therapy-treated section demonstrating a weak positive expression of VEGF (VEGF immunostain, counterstained with hematoxylin). **(f)** Statistical analysis of VEGF cytoplasmic expression levels in the skeletal muscle sections of the different mice experimental groups. The data are presented as the mean ± SD of three independent biological replicates per group. Different letters ^(a, b, and c)^ demonstrate significant differences (*p* ≤ 0.05, one-way ANOVA).

**Fig. 10:**
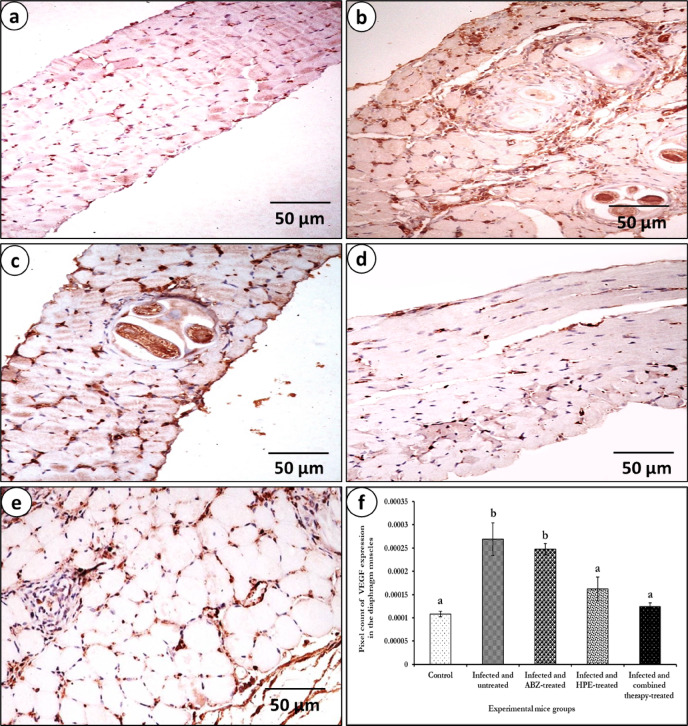
Photomicrographs of vascular endothelial growth factor (VEGF) cytoplasmic expression levels appeared as a brown color in the diaphragm muscle sections of mice: **(a)** a control section demonstrating a weak positive VEGF expression, **(b)** an infected, untreated section demonstrating a strong positive VEGF expression primarily in the cytoplasm of the inflammatory cells between diaphragmatic muscle fibers and around nurse cells, **(c)** an infected and ABZ-treated section demonstrating a moderate positive expression of VEGF, **(d)** an infected and HPE-treated section demonstrating a mild positive expression of VEGF, and **(e)** an infected and combined therapy-treated section demonstrating a weak positive expression of VEGF (VEGF immunostain, counterstained with hematoxylin). **(f)** Statistical analysis of VEGF cytoplasmic expression levels in the diaphragm muscle sections of the different mice experimental groups. The data are presented as the mean ± SD of three independent biological replicates per group. Different letters ^(a, b, and c)^ demonstrate significant differences (*p* ≤ 0.05, one-way ANOVA).

Immunohistochemical analysis of VEGF demonstrated highly significant increases (*p* < 0.001) in its expression levels in both skeletal and diaphragmatic muscles of the infected, untreated group compared with the control group. Treatment with ABZ resulted in non-significant reductions (*p* > 0.05) in VEGF expression in both muscle types compared with the infected, untreated group, and VEGF expression remained significantly higher (*p* ≤ 0.001) than that of the control group. HPE treatment significantly reduced VEGF expression levels in both skeletal and diaphragmatic muscles (*p* < 0.01 and *p* = 0.001, respectively) compared with the infected, untreated group, with skeletal muscles showing a significant increase (*p* < 0.05) relative to the control group, while diaphragmatic muscle showed no significant difference (*p* > 0.05) from the control group. The combined therapy produced the greatest reduction in VEGF expression in both muscle types, showing highly significant decreases (*p* < 0.001) compared with the infected, untreated group and no significant differences (*p* > 0.05) compared with the control group (Figs. 9 and 10 and Table S4).

## Discussion

Adverse effects and emerging drug resistance remain major limitations of current anti-*Trichinella* therapies^48^, emphasizing the need for alternative treatment approaches. Previous studies have reported anti-*Trichinella* larval activity of various plant-derived compounds^7,49,50^; however, the potential effects of marine invertebrates on encysted larvae have not yet been explored. Therefore, this study evaluated the therapeutic efficacy of *H. polii* ethanol extract against the muscular phase of *T. spiralis* infection and its potential adjunctive effect with ABZ. Male mice were used to minimize sex-related hormonal and immunological variability that could influence infection progression and treatment response. This approach is supported by Hasby et al.^51^, who demonstrated that hormonal modulation significantly affected parasite burden and host immune responses in experimental *T. spiralis* infection.

The parasitological findings confirmed an adequate infection level, consistent with Albogami^52^, allowing a reliable evaluation of therapeutic efficacy. All treated groups showed significant reductions in muscle larval counts compared with the infected, untreated group. The combined therapy achieved the highest larval reduction (83.33%), followed by HPE (68.47%) and ABZ (54.04%). The lower efficacy of ABZ against encysted larvae agrees with Hamed et al.’s results^53^ (56.49%) and is attributed to its poor water solubility and low bioavailability^54^. Notably, the combined therapy demonstrated strong larvicidal activity (83.33%), close to that reported by El-Saidy et al.^26^ against adult worms (96.76%), indicating effectiveness against both adult and larval stages of *T. spiralis*. The antiparasitic effect of HPE is likely related to its rich content of bioactive compounds, particularly triterpene glycosides and other constituents previously reported to possess anthelmintic activity^26,28,32,55^.

During *T. spiralis* infection, excessive ROS and free radical production induce lipid peroxidation, reflected by elevated MDA levels and depletion of antioxidant defenses such as GSH level and CAT activity, which normally scavenge free radicals and prevent membrane damage^13,56,57^. In the current work, infected, untreated mice exhibited significantly increased MDA levels and decreased GSH levels and CAT activities in skeletal muscles compared with control mice, indicating oxidative imbalance consistent with previous findings^13,58^, likely due to antioxidant exhaustion during ROS scavenging^59^. Treatment with either ABZ or HPE alone, and more markedly their combination, significantly improved antioxidant status and reduced lipid peroxidation versus infected, untreated mice; however, only combined therapy restored values close to control levels, indicating stronger attenuation of oxidative stress. This improvement is attributed to reduced larval burden and restoration of redox balance, as reported by Albogami^52^, in addition to the antioxidant phytochemicals in HPE (saponins, phenolics, alkaloids, and flavonoids) and their synergism with ABZ^26^. These phytochemicals exert free radical scavenging activity and inhibit lipid oxidation^60–62^.

*T. spiralis* infection induces liver damage as a secondary effect of the host’s immune response to migrating larvae and infection-related oxidative stress and systemic inflammation, leading to hepatic lesions and dysfunction^63^. Muscle damage mainly results from direct larval invasion and encystment within muscle fibers^8^. Serum liver and muscle enzymes are widely used to evaluate infection severity and treatment efficacy during the muscular phase^64^. ALT is a specific marker of hepatocellular injury, whereas AST reflects both hepatic and muscular damage due to its high activity in these tissues^65,66^. ALP indicates plasma membrane integrity, and its altered activity level reflects membrane damage^67^. Additionally, elevated serum LDH and CK activities are indicators of muscle injury, with CK being closely associated with skeletal muscle destruction caused by *Trichinella* larval invasion^68,69^.

The present study demonstrated highly significant elevations in serum ALT, AST, ALP, LDH, and CK activities in infected, untreated mice relative to control mice, indicating hepatic and muscular damage induced by *T. spiralis* infection. These findings align with previous studies reporting elevated ALT, AST, LDH, and CK activity levels following *T. spiralis* infection^64,66,70^, which were associated with larval burden and parasite-induced tissue injury, as enzyme elevations were directly proportional to the number of inoculated larvae^71^. Treatment with either ABZ or HPE, alone or in combination, significantly reduced the activities of all investigated serum enzymes relative to infected, untreated mice, with the combined therapy showing the strongest normalization effect. The hepatoprotective and myoprotective effects of HPE may be linked to its antioxidant saponins and polyphenols. This is supported by studies showing improvements in liver function markers, ALT, AST, and ALP, following treatment with saponins extracted from *Panax japonica* and *Astragalus*^72,73^, while polyphenols may account for decreases in serum LDH and CK activities^74^. Additionally, Shalaby et al.^75^ reported reduced serum activity levels of AST and CK following treatment with *Balanites aegyptiaca* extract, likely through antioxidant-mediated protection against *T. spiralis*-induced tissue damage.

Histopathological examination of skeletal and diaphragmatic muscles in infected, untreated mice revealed severe architectural disruption, with many *T. spiralis* encysted larvae, accompanied by marked inflammatory infiltrations around the capsules and between degenerated muscle fibers, consistent with previous reports of Elmehy et al.^14^ and El-Wakil et al.^49^. Larval-induced ROS production and collagen capsule formation contribute to persistent inflammation and myocyte damage^76,77^. ABZ treatment reduced larval burden, as evidenced by fewer encysted larvae in both muscle tissues than those observed in the infected, untreated mice; however, partially degenerated capsules with inflammation and muscle fiber separation persisted, likely due to poor solubility and limited bioavailability of ABZ^78^. In contrast, HPE improved muscle architecture with mild inflammation, while combined therapy showed the most pronounced effect, preserving muscular tissue architecture with degenerated larval capsules and minimal inflammatory infiltrates, attributed to the anti-inflammatory capacity of HPE^31^, linked to its anti-inflammatory constituents, including saponins, phenolics, alkaloids, and flavonoids^79–82^.

COX-2 is an inducible pro-inflammatory enzyme upregulated by cytokines like tumor necrosis factor-alpha, interleukin-1, and interleukin-17, resulting in increased prostaglandin E₂ synthesis and inflammation^83–85^. *T. spiralis* larvae induce angiogenesis around nurse cells, which is primarily associated with upregulation of VEGF, facilitating nutrient delivery and waste removal needed for larval survival^9^. In the current study, infected, untreated mice showed a significant increase in COX-2 and VEGF cytoplasmic expressions in both skeletal and diaphragmatic muscles, mainly within inflammatory cells between muscle fibers and around nurse cells, compared with control mice. These findings are consistent with the results of El-Aswad et al.^86^, who reported a strong positive expression of COX-2 in the skeletal muscles of *T. spiralis*-infected mice at the 35^th^ dpi, and Elgendy et al.^58^, who reported a strong positive expression of VEGF in the *T. spiralis*-infected group. On the other hand, treatment with ABZ resulted in moderate positive COX-2 expression levels, while HPE treatment showed moderate to mild positive COX-2 and VEGF expression levels in sections of skeletal and diaphragmatic muscle fibers, respectively. Notably, combined therapy markedly reduced COX-2 and VEGF expression levels to near-control expression levels, with weak positive expression levels in sections of both skeletal and diaphragmatic muscles, showing no significant differences from control mice. The reduction in COX-2 expression indicates effective suppression of muscle inflammation, consistent with Lu et al.^87^, who reported that COX-2 upregulation is associated with inflammation, whereas its reduction reflects attenuation of inflammatory processes within the muscles. The observed reductions in VEGF expression levels in treated groups suggest inhibition of angiogenesis, which may compromise larval survival by limiting access to nutrients and impairing waste disposal^34^. Anti-angiogenic action is likely owing to the high saponin content of HPE, as saponins are known to suppress angiogenesis^88^. Although the present study demonstrated significant modulation of inflammatory and angiogenic markers by HPE, the underlying molecular pathways responsible for these effects were not fully elucidated and warrant further investigation.

## Conclusion

HPE exhibited an anti-trichinellosis effect by reducing *T. spiralis* larval burden and ameliorating oxidative stress in infected tissues. It improved antioxidant status and normalized serum activity levels of liver and muscle enzymes. Histopathological and immunohistochemical findings revealed reduced tissue damage and downregulation of inflammatory and angiogenic marker expressions. The combined treatment achieved the highest efficacy, supporting HPE as a potential complementary treatment to ABZ against trichinellosis. However, further studies are recommended to purify the bioactive constituents of HPE and to elucidate their molecular mechanisms and their potential efficacy in clinical applications against trichinellosis.

## Supplementary Information

Below is the link to the electronic supplementary material.

Supplementary Material 1

## Data Availability

All data generated or analyzed during this study are included in this published article.

## Abbreviations

ABZ: Albendazole
ALP: Alkaline phosphatase
ALT: Alanine aminotransferase
ANOVA: Analysis of variance
AST: Aspartate aminotransferase
b.w.: Body weight
C: Capsule
CAT: Catalase
CK: Creatine kinase
COX-2: Cyclooxygenase-2
dpi: day post-infection
GSH: Reduced glutathione
H&E: Hematoxylin and eosin
H₂O₂: Hydrogen peroxide
HPE: *Holothuria polii* extract
L: Larvae
LDH: Lactate dehydrogenase
LI: Leucocytic infiltration
MDA: Malondialdehyde
NC: Nurse cell
PBS: Phosphate-buffered saline
ROS: Reactive oxygen species
SD: Standard deviation
Tukey’s HSD: Tukey’s Honest Significant Difference
VEGF: Vascular endothelial growth factor
