# Supplementary material for: Assessment of the therapeutic efficacy of Holothuria polii extract during the muscular phase of Trichinella spiralis infection in albino mice
Source: Sci Rep. 2026 Jul 5;16:20567. doi: 10.1038/s41598-026-60075-y (PMC13333944; doi:10.1038/s41598-026-60075-y)
Supplement: Supplementary file 1 — Supplementary Material 1 [file 41598_2026_60075_MOESM1_ESM.doc]

**Assessment of the therapeutic efficacy of *Holothuria polii* extract during the muscular phase of *Trichinella spiralis* infection in albino mice**

**Salwa A. El-Saidy 1,*, Asmaa Sabry 1, Gihan M. El-Khodary 1, Amal A. A. Hassan 1,** **Dina I. Elgendy 2, and Yasmeen M. Gawaan 1**

1 Zoology Department, Faculty of Science, Damanhour University, Damanhour, Egypt

2 Medical Parasitology Department, Faculty of Medicine, Tanta University, Tanta, Egypt

* Corresponding Author: Salwa A. El-Saidy, Zoology Department, Faculty of Science, Damanhour University, Damanhour, Postal code: 22511, Egypt. +201008025674, [s.elsaidy@sci.dmu.edu.eg](mailto:s.elsaidy@sci.dmu.edu.eg)

**Table S1.** Count of *T. spiralis* larvae in the muscles of the different infected mice groups.

| **Groups** | **Larval count**  **(larvae/mouse)** | **Reduction (%)** | **F-value** | ***p*-value** |
| --- | --- | --- | --- | --- |
| **Infected and untreated** | 7399.33 ± 162.20d | **-** | 1106.70 | 0.00*** |
| **Infected and ABZ-treated** | 3400 ± 136c | 54.04 |
| **Infected and HPE-treated** | 2333 ± 124.89b | 68.47 |
| **Infected and combined therapy-treated** | 1233.33 ± 134a | 83.33 |

The data are presented as the mean ± SD of three independent biological replicates per group. Reduction percentages were calculated relative to the infected, untreated group. Means in the same column with different superscript letters (a, b, c, and d) demonstrate significant differences (*p* ≤ 0.05) based on a one-way ANOVA test, followed by Tukey’s HSD test. ***: Asterisks denote significant differencesat *p* ≤ 0.001.

**Table S2.** Oxidative stress and antioxidant markers in the skeletal muscular tissue of the different mice groups.

| **Groups** | **MDA**  **(nmol/g tissue)** | **Reduction (%)** | **GSH**  **(mg/g tissue)** | **Increase (%)** | **CAT**  **(U/g tissue)** | **Increase (%)** |
| --- | --- | --- | --- | --- | --- | --- |
| **Control** | 105.33 ± 1.52a | **-** | 53.66 ± 1.10c | - | 51.73 ± 4d | - |
| **Infected and untreated** | 142.66 ± 2.51c | **-** | 32.20 ± 1.96a | - | 19.66 ± 1.68a | - |
| **Infected and ABZ-treated** | 128 ± 2b | 10.27 | 42.03 ± 1.85b | 30.52 | 29.50 ± 1.86b | 50.05 |
| **Infected and HPE-treated** | 123.33 ± 1.52b | 13.54 | 45.66 ± 1.19b | 41.80 | 39.83 ± 3.16c | 102.59 |
| **Infected and combined therapy-treated** | 109 ± 1a | 23.59 | 50.06 ± 1.83c | 55.46 | 46.40 ± 1.60cd | 136.01 |
| **F-value** | 213.54 |  | 77.09 |  | 71.72 |  |
| ***p*-value** | 0.00*** |  | 0.00*** |  | 0.00*** |  |

Malondialdehyde (MDA), reduced glutathione (GSH), and catalase (CAT). The data are presented as the mean ± SD of three independent biological replicates per group. Change percentages were calculated relative to the infected, untreated group. Means in the same column with different superscript letters (a, b, c, and d) demonstrate significant differences (*p* ≤ 0.05) based on a one-way ANOVA test, followed by Tukey’s HSD test. ***: Asterisks denote significant differencesat *p* ≤ 0.001.

**Table S3. Serum enzymatic activities in the different mice groups.**

| **Groups** | **ALT**  **(U/L)** | **Reduction (%)** | **AST**  **(U/L)** | **Reduction (%)** | **ALP**  **(U/L)** | **Reduction (%)** | **LDH**  **(U/L)** | **Reduction (%)** | **CK**  **(U/L)** | **Reduction (%)** |
| --- | --- | --- | --- | --- | --- | --- | --- | --- | --- | --- |
| **Control** | 30 ± 1a | **-** | 149.33 ± 4.04a | - | 21.33 ± 1.52a | - | 2848.66 ± 11.50a | - | 91.66 ± 3.05a | - |
| **Infected and untreated** | 63.66 ± 4.16c | **-** | 258 ± 2.64d | - | 46 ± 3.60 c | - | 3679.33 ± 23.24d | - | 623.33 ± 25.16d | - |
| **Infected and ABZ-treated** | 41 ± 3.60 b | 35.59 | 195 ± 5c | 24.41 | 34.33 ± 2.08b | 25.36 | 3395 ± 15.52c | 7.72 | 425.33 ± 23.35c | 31.76 |
| **Infected and HPE-treated** | 37 ± 2.64ab | 41.87 | 163.33 ± 3.78b | 36.69 | 25.33 ± 2.08a | 44.93 | 3003.66 ± 20.50b | 18.36 | 263 ± 13b | 57.80 |
| **Infected and combined therapy-treated** | 32.66 ± 2.51a | 48.69 | 159 ± 3.60ab | 38.37 | 23.66 ± 1.52a | 48.56 | 2860.33 ± 15.30a | 22.25 | 131 ± 3.60a | 78.98 |
| **F-value** | 60.45 |  | 388.87 |  | 58.62 |  | 1281.77 |  | 529.24 |  |
| ***p*-value** | 0.00*** |  | 0.00*** |  | 0.00*** |  | 0.00*** |  | 0.00*** |  |

Alanine aminotransferase (ALT), aspartate aminotransferase (AST),alkaline phosphatase (ALP), lactate dehydrogenase (LDH),andcreatine kinase (CK). The data are presented as the mean ± SD of three independent biological replicates per group. Reduction percentages were calculated relative to the infected, untreated group. Means in the same column with different superscript letters (a, b, c, and d) demonstrate significant differences (*p* ≤ 0.05) based on a one-way ANOVA test, followed by Tukey’s HSD test. ***: Asterisks denote significant differencesat *p* ≤ 0.001.

**Table S4. Statistical analysis of cytoplasmic expression levels of immunohistochemical markers in the muscle sections of the different mice groups after ImageJ analysis.**

| **Groups** | **COX-2 expression levels** | | | | **VEGF expression levels** | | | |
| --- | --- | --- | --- | --- | --- | --- | --- | --- |
|  | **Skeletal muscle sections (Pixels)** | **Reduction (%)** | **Diaphragm muscle sections (Pixels)** | **Reduction (%)** | **Skeletal muscle sections (Pixels)** | **Reduction (%)** | **Diaphragm muscle sections (Pixels)** | **Reduction (%)** |
| **Control** | 0.000117 ± 0.000018a | - | 0.000112 ± 0.000009a | - | 0.000130 ± 0.000022a | - | 0.000108 ± 0.000006a | - |
| **Infected and untreated** | 0.000289 ± 0.000000016643c | - | 0.000281 ± 0.000004b | - | 0.000286 ± 0.000003c | - | 0.000269 ± 0.000035b | - |
| **Infected and ABZ-treated** | 0.000234 ± 0.000019bc | 19.03 | 0.000248 ± 0.00002b | 11.74 | 0.000244 ± 0.000021bc | 14.68 | 0.000248 ± 0.000012b | 7.80 |
| **Infected and HPE-treated** | 0.000185 ± 0.000043b | 35.98 | 0.000176 ± 0.000048a | 37.36 | 0.000203 ± 0.000035b | 29.02 | 0.000162 ± 0.000026a | 39.77 |
| **Infected and combined therapy-treated** | 0.000119 ± 0.000009a | 58.82 | 0.000125 ± 0.000008a | 55.51 | 0.000132 ± 0.000016a | 53.84 | 0.000124 ± 0.000008a | 53.90 |
| **F-value** | 31.54 |  | 29.51 |  | 29.35 |  | 37.30 |  |
| ***p*-value** | 0.00*** |  | 0.00*** |  | 0.00*** |  | 0.00*** |  |

Cyclooxygenase-2 (COX-2) and vascular endothelial growth factor (VEGF). The data are presented as the mean ± SD of three independent biological replicates per group. Reduction percentages were calculated relative to the infected, untreated group. Means in the same column with different superscript letters (a, b, and c) demonstrate significant differences (*p* ≤ 0.05) based on a one-way ANOVA test, followed by Tukey’s HSD test. ***: Asterisks denote significant differencesat *p* ≤ 0.001.
